# A genome-wide association and fine-mapping study of white rust resistance in hexaploid chrysanthemum cultivars with a wild diploid reference genome

**DOI:** 10.1093/hr/uhac170

**Published:** 2022-08-03

**Authors:** Katsuhiko Sumitomo, Kenta Shirasawa, Sachiko Isobe, Hideki Hirakawa, Akiho Harata, Michiharu Nakano, Yoshihiro Nakano, Masafumi Yagi, Tamotsu Hisamatsu, Hiroyasu Yamaguchi, Fumiya Taniguchi

**Affiliations:** Institute of Vegetable and Floriculture Science, NARO, Tsukuba, Ibaraki 305-0852, Japan; Kazusa DNA Research Institute, Kisarazu, Chiba 292-0818 Japan; Kazusa DNA Research Institute, Kisarazu, Chiba 292-0818 Japan; Kazusa DNA Research Institute, Kisarazu, Chiba 292-0818 Japan; Kagoshima Prefectural Institute for Agricultural Development, Minamisatsuma, Kagoshima 899-3401, Japan; CCS Inc., Kyoto, Kyoto 602-8019, Japan; Graduate School of Integrated Sciences for Life, Hiroshima University, Higashi-Hiroshima, Hiroshima 739-8526, Japan; Faculty of Agriculture and Marine Science, Kochi University, Nankoku, Kochi 783-8502, Japan; Institute of Vegetable and Floriculture Science, NARO, Tsukuba, Ibaraki 305-0852, Japan; Institute of Vegetable and Floriculture Science, NARO, Tsukuba, Ibaraki 305-0852, Japan; Institute of Vegetable and Floriculture Science, NARO, Tsukuba, Ibaraki 305-0852, Japan; Institute of Vegetable and Floriculture Science, NARO, Tsukuba, Ibaraki 305-0852, Japan; Institute of Fruit Tree and Tea Science, NARO, Tsukuba, Ibaraki 305-8605, Japan

## Abstract

White rust caused by *Puccinia horiana* is one of the most serious diseases of chrysanthemum (*Chrysanthemum* × *morifolium*). In this study, we report the DNA markers associated with resistance against *P. horiana* via a simple approach using the genome of a wild diploid relative, *Chrysanthemum seticuspe*. First, we identified the important region of the genome in the resistant cultivar “Ariesu” via a genome-wide association study. Simplex single nucleotide polymorphism (SNP) markers mined from ddRAD-Seq were used in a biparental population originating from crosses between resistant “Ariesu” and susceptible “Yellow Queen”. The *C. seticuspe* genome was used as a reference. For the fine mapping of *P. horiana* resistance locus 2 (*Phr2*), a comparative whole genome sequencing study was conducted. Although the genome sequences of chrysanthemum cultivars assembled via the short-read approach were fragmented, reliable genome alignments were reconstructed by mapping onto the chromosome level of the *C. seticuspe* pseudomolecule. Base variants were then identified by comparing the assembled genome sequences of resistant “Ariesu” and susceptible “Yellow Queen”. Consequently, SNP markers that were closer to *Phr2* compared with ddRAD-Seq markers were obtained. These SNP markers co-segregated with resistance in F_1_ progenies originating from resistant “Ariesu” and showed robust transferability for detecting *Phr2*-conferring resistance among chrysanthemum genetic resources. The wild *C. seticuspe* pseudomolecule, a *de facto* monoploid genome used for ddRAD-Seq analysis and assembled genome sequence comparison, demonstrated this method’s utility as a model for developing DNA markers in hexaploid chrysanthemum cultivars.

## Introduction

Chrysanthemum (*Chrysanthemum* × *morifolium* Ramat.) is an important ornamental crop worldwide. White rust is a major disease caused by *Puccinia horiana* Henn. in chrysanthemums. It was first detected in Japan in 1895 [3, 21]. Since then, it has spread worldwide [35]. *P. horiana* forms raised blisters or pink pustules primarily on the lower surface of the leaves, causing major economic losses in commercial production. One of the most effective disease control methods is the use of *P. horiana*-resistant cultivars. Several *P. horiana*-resistant chrysanthemum cultivars have been reported [3, 7–9, 31, 38, 51]. Therefore, developing resistance-linked DNA markers will contribute to efficient resistance breeding.

To date, DNA marker development for white rust has been challenging because chrysanthemum is a highly heterozygous outcrossing hexaploid species with many chromosomes (2*n* = 6*x* = 54), which complicates genetic analyses. However, this problem is now being resolved using massive parallel sequencing technology. For example, van Geest *et al*. [17] showed that chrysanthemum’s inheritance mode is hexasomic, i.e. random chromosome pairing occurs during bivalent formation. Furthermore, quantitative trait loci have been detected using highly sophisticated methods that can handle genome complexity [16]. Moreover, *Chrysanthemum seticuspe*, a wild species of diploid chrysanthemum for which whole genome assembly has been performed previously, is established as a model strain in the genus *Chrysanthemum* [20, 33, 34]. Thus, the diploid *C. seticuspe* genome has been used as a reference for efficiently developing DNA markers in chrysanthemum cultivars [45]. Using the genome sequence of *C. seticuspe* as a reference for mining DNA polymorphisms from ddRAD-Seq data of hexaploid cultivated chrysanthemum, DNA markers linked to the resistance genetic locus *Phr1* have been reported [46]. According to gene-for-gene interactions [14, 15], plants have evolved sophisticated resistance systems against pathogens, whereas pathogens have evolved functions by which they evade these plant resistance systems. Through these interactions, plants have acquired many resistance genes. Based on this theory, chrysanthemum may have acquired multiple resistance genes against *P. horiana*. Previous inoculation experiments [7, 51] indicated that more useful resistance genes besides *Phr1* likely exist in chrysanthemum cultivars. Identifying the molecular markers associated with other resistance genes will contribute to marker-assisted selection and promote systematic resistance breeding (e.g. pyramiding resistance genes).

This study aimed to develop a highly accurate DNA marker for a novel *P. horiana* resistance locus to aid marker-assisted selection and resistance detection. Previously, ddRAD-Seq analysis was used to develop chrysanthemum DNA markers [45, 46]. This approach is feasible for species with high heterozygosity and large genomes, such as chrysanthemum cultivars [13]. In ddRAD-Seq analysis, DNA polymorphisms are identified from restriction-site associated sequences. Thus, linkage between DNA markers and traits of interest is sometimes weak due to the genome complexity reduction. In addition, a single biparental mapping population might be insufficient to saturate DNA markers on a linkage map if genetic distance between the parents is close. Consequently, to design highly accurate DNA markers, obtaining information on sequences that exist in the gaps between ddRAD-Seq markers is necessary. Unfortunately, whole genome alignments of cultivated chrysanthemums are underdeveloped. Long-read sequencing technologies are currently the most powerful approach for obtaining unknown gap sequences; however, they are not cost-effective because the whole genome size of cultivated chrysanthemum is estimated to be as large as approximately 18 Gbp by flow cytometry [32]. Alternatively, short-read sequencing analysis can provide a large amount of data at a relatively low cost; thus, mapping such reads to the genome of a diploid wild species may offer a solution to this problem. Recently, a pseudomolecule of *C. seticuspe* was reported (http://mum-garden.kazusa.or.jp). Accordingly, using the genome sequence of *C. seticuspe* as a reference, we conducted whole genome sequencing and identified the sequence information needed to develop accurate DNA markers for chrysanthemum cultivars. We also tested the transferability and use of the resulting DNA markers in the genetic detection of *P. horiana* resistance.

## Results

### *Inheritance of* P. horiana *resistance in “Ariesu”*

**Figure 1:**
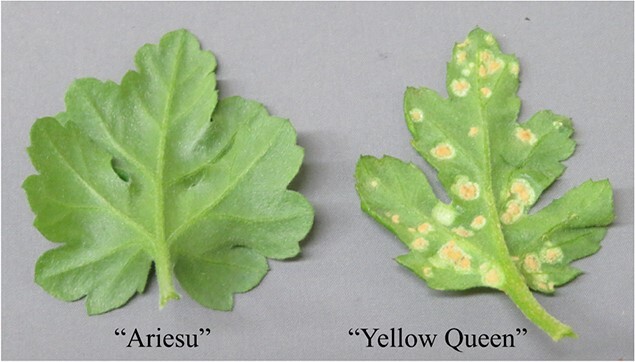
Abaxial sides of leaves of resistant “Ariesu” and susceptible “Yellow Queen”, respectively, at 35 days after inoculating the *P. horiana* isolate TS.

The reaction of “Ariesu” and “Yellow Queen” to *P. horiana* isolates was investigated using the inoculation assay. “Ariesu” exhibited resistance to six isolates (TS, AK, IB, TO1, TO2 and TO3) but was susceptible to NA (Table 1; Fig. 1). “Yellow Queen” exhibited susceptibility to all isolates. Next, the reaction of the 283 F_1_ individuals originating from reciprocal crosses between “Ariesu” and “Yellow Queen” to the *P. horiana* isolate TS was investigated. The F_1_ individuals were segregated with a resistance:susceptibility ratio of 145:138. This result was close to the expected ratio of 1:1 (χ^2^ = 0.17; *p* = 0.68) of a simplex × nulliplex (Aaaaaa × aaaaaa) cross for hexasomic inheritance, following the chrysanthemum segregation pattern. From the F_1_ population, eight resistant individuals and eight susceptible individuals were selected to isolate TS, and an inoculation assay was conducted using the other six isolates. All eight individuals showing resistance to TS also exhibited resistance against AK, IB, TO1, TO2 and TO3 but were susceptible to NA (Table 1; Supplementary Figure S1). The interaction phenotype profile was consistent with that of “Ariesu”. In contrast, all eight individuals that were susceptible to TS also exhibited susceptibility to the other six isolates. These results indicated that “Ariesu” had a single dominant genetic locus, i.e. *Phr2*, effective against six isolates.

**Table 1:** Reaction of “Ariesu”, “Yellow Queen” and the representative F_1_ plants to seven *P. horiana* isolates. Plants that showed no telia were scored as “R”. Plants were scored as “S” when at least one telium was observed on a plant. *P. horiana* resistance was examined by three separate inoculations per isolate

Cultivar and F_1_ individual	Reaction to *P. horiana* isolates						
	TS	NA	AK	IB	TO1	TO2	TO3
Ariesu	R	S	R	R	R	R	R
Yellow Queen	S	S	S	S	S	S	S
YA-01	R	S	R	R	R	R	R
YA-03	R	S	R	R	R	R	R
YA-05	R	S	R	R	R	R	R
YA-07	R	S	R	R	R	R	R
YA-10	R	S	R	R	R	R	R
YA-14	R	S	R	R	R	R	R
YA-25	R	S	R	R	R	R	R
YA-32	R	S	R	R	R	R	R
YA-04	S	S	S	S	S	S	S
YA-09	S	S	S	S	S	S	S
YA-11	S	S	S	S	S	S	S
YA-26	S	S	S	S	S	S	S
YA-27	S	S	S	S	S	S	S
YA-29	S	S	S	S	S	S	S
YA-37	S	S	S	S	S	S	S
YA-43	S	S	S	S	S	S	S

### *GWAS and linkage analysis for* P. horiana *resistance in “Ariesu”*

Via GWAS, DNA markers associated with resistance in “Ariesu” were developed using biparental F_1_ populations. Approximately 2.1 M high-quality reads per sample were obtained. Of these sequence reads, 82.9% were mapped onto the *C. seticuspe* reference genome, CSE_r1.0 [20]. In total, 274 700 SNP candidates were obtained. After filtering with criteria of number of reads (≥10) and missing data (<0.75), 73 025 high-confidence SNP candidates were identified. Of these, 1598 double-simplex (Aaaaaa × Aaaaaa or AAAAAa × AAAAAa) and 9181 simplex (Aaaaaa × aaaaaa, aaaaaa × Aaaaaa, AAAAAa × AAAAAA or AAAAAA × AAAAAa) SNPs were selected.

The generalised linear model analysis of 10 779 (i.e. 1598 + 9181) SNP markers identified 82 SNP markers that were significantly associated with *P. horiana* resistance (Supplementary data Table S4). The SNP marker SCSE_SC004884.1_65872 exhibited the highest association with the lowest *p*-value of 1.12 × 10^−113^. The ddRAD-Seq results showed that the genotypes of the SNP marker SCSE_SC004884.1_65872 in resistant “Ariesu” were TTTTTC, whereas those in susceptible “Yellow Queen” were TTTTTT. Additionally, the genotypes of SCSE_SC000716.1_75925, which had the second-lowest *p*-value of 2.76 × 10^−106^, were AAAAAG in “Ariesu” and AAAAAA in “Yellow Queen”. The minor C allele of SCSE_SC004884.1_65872 and the minor G allele of SCSE_SC000716.1_75925 were in the coupling phase of the resistant allele in *Phr2* (Table 2).

**Table 2:** Relationship between marker genotypes and *P. horiana* resistance in 283 F_1_ plants obtained from reciprocal crosses between “Ariesu” and “Yellow Queen”

Marker genotype^a^		*P. horiana* resistance^b^	
SCSE_SC004884.1_65872	SCSE_SC000716.1_75925	Resistant	Susceptible
TTTTTC	AAAAAG	142	0
TTTTTC	AAAAAA	0	1
TTTTTT	AAAAAG	3	6
TTTTTT	AAAAAA	0	131

a The genotypes of SCSE_SC004884.1_65872 in resistant “Ariesu” and susceptible “Yellow Queen” were TTTTTC and TTTTTT, respectively. The genotypes of SCSE_SC000716.1_75925 in resistant “Ariesu” and susceptible “Yellow Queen” were AAAAAG and AAAAAA, respectively.

b Plants that showed no telia were scored as resistant. Plants were scored as susceptible when at least one telium was observed. *P. horiana* resistance was examined by three separate inoculations using the isolate TS.

### Fine mapping by comparing “Ariesu” and “Yellow Queen” whole genome sequences

**Figure 2:**
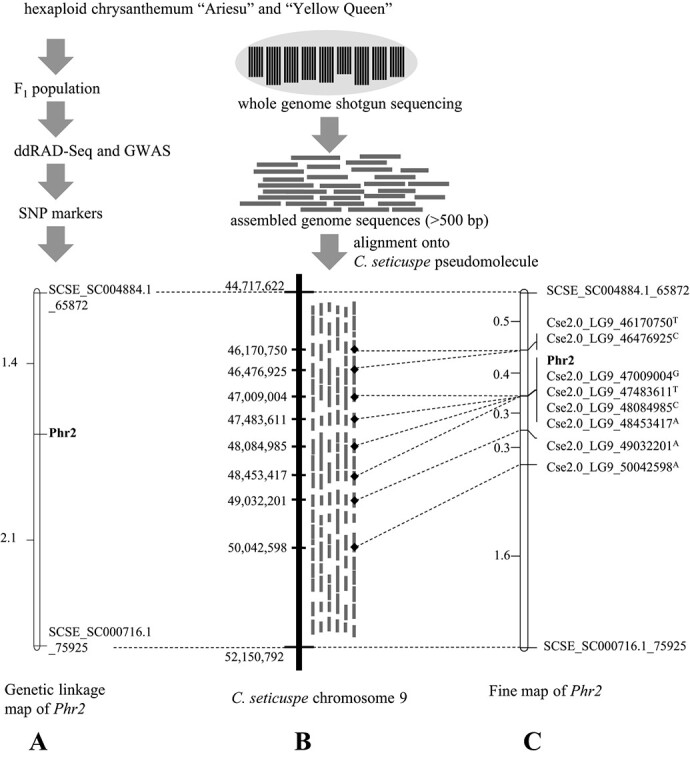
Overview of the process for developing single nucleotide polymorphism (SNP) markers and fine mapping for *Phr2* locus with a combination of genome-wide association study and whole genome sequencing in chrysanthemum cultivars using the genome of a wild species, diploid *C. seticuspe*, as a reference. (A) Partial linkage map containing the *Phr2* locus. The SNP markers SCSE_SC004884.1_65872 and SCSE_SC000716.1_75925 were obtained by ddRAD-Seq analysis and association study in the F_1_ biparental population derived from reciprocal crosses between “Ariesu” and “Yellow Queen” (n = 283). Genetic distances (cM) are indicated on the left. (B) Assembled genome sequences of “Ariesu” and “Yellow Queen” (short grey lines) mapped onto the *C. seticuspe* chromosome (bold line) for identifying SNP markers between ddRAD-Seq markers. The corresponding positions of the markers were determined on the *C. seticuspe* genome sequence of chromosome 9 (CSE_r2.0, http://mum-garden.kazusa.or.jp). Positions (bp) are indicated on the left. (C) Fine map of *Phr2* generated from the F_1_ biparental population derived from reciprocal crosses between “Ariesu” and “Yellow Queen” (n = 656) using SNP markers obtained by comparing the assembled genome sequences of parents. Genetic distances (cM) and marker names are indicated on the left and right, respectively.

To obtain markers closely linked to the *Phr2* locus, we analysed the sequences of genomic regions between the two markers SCSE_SC004884.1_65872 and SCSE_SC000716.1_75925 and designed primer pairs. Linkage analysis indicated that *Phr2* was located at a genetic distance of 1.4 cM from SCSE_SC004884.1_65872 and 2.1 cM from SCSE_SC000716.1_75925 (Fig. 2A). The genome of cultivated chrysanthemums has not yet been elucidated; thus, the sequence between the two SNP markers is unclear. Therefore, we assumed that the pseudomolecule of diploid *C. seticuspe* could act as a reference genome for cultivated chrysanthemum. The base sequences associated with the SNP markers SCSE_SC004884.1_65872 and SCSE_SC000716.1_75925 were then positioned on chromosome 9 of the *C. seticuspe* pseudomolecule. This region spanned a physical interval of ~7.4 Mbp (Fig. 2B; SCSE_SC004884.1_65872 to SCSE_SC000716.1_75925: 44717622–52 150 792 bp).

SOAPdenovo2 was used to assemble 789.0- and 880.1-Gbp HiSeq paired-end reads of the whole “Ariesu” and “Yellow Queen” genomes (k-mer = 101), respectively. Overall, 101 290 730 and 97 821 561 contigs of “Ariesu” and “Yellow Queen”, respectively, were generated (Supplementary data Table S5), with the assembled genomes showing ultrafragmentation. The total and N50 lengths were 21 001 896 460 and 203 bp in “Ariesu” and 20 449 854 573 and 203 bp in “Yellow Queen”, respectively. We selected 3 743 825 and 3 521 557 contigs that were > 500 bp in size of “Ariesu” and “Yellow Queen”, respectively (Supplementary data Table S5; accession numbers BOUO010000001–BOUO013743825 for “Ariesu” and BOUP010000001–BOUP013521557 for “Yellow Queen”), mapped these onto the *C. seticuspe* pseudomolecule and finally obtained the discontinuous genome sequences of “Ariesu” and “Yellow Queen” (Fig. 2B). Eight SNP markers were designed every 0.5 Mbp in the 46.1–50.0-Mbp region on chromosome 9 of the *C. seticuspe* pseudomolecule (Table 3).

These SNP markers were used to delimit the *Phr2* locus with more precision. Of 656 F_1_ plants, we identified 20 F_1_ recombinant plants exhibiting genetic recombination between SCSE_SC004884.1_65872 and SCSE_SC000716.1_75925. Subsequently, we analysed the genotypes of these 20 plants with the 8 SNP markers. The *Phr2* locus was narrowed to a 0.7-cM genetic interval between the SNP markers Cse2.0_LG9_46170750^T^, Cse2.0_LG9_46476925^C^ and Cse2.0_LG9_49032201^A^ (Fig. 2C). The potential region between these markers in “Ariesu” should contain the gene conferring resistance and correspond to a region of ~2.6 Mbp (Cse2.0_LG9_46476925^C^ to Cse2.0_LG9_49032201^A^: 46476925–49 032 201 bp) on chromosome 9 of the *C. seticuspe* pseudomolecule.

### *Markers for detecting* P. horiana *resistance*

Four SNP markers (Cse2.0_LG9_47009004^G^, Cse2.0_LG9_47483611^T^, Cse2.0_LG9_48084985^C^ and Cse2.0_LG9_48453417^A^) co-segregated with *Phr2* in the 656 F_1_ progenies (Fig. 2C), suggesting that they were in linkage disequilibrium with *Phr2* and that cultivars with *Phr2* resistance-conferring genes could be identified among the chrysanthemum genetic resources. To assess transferability, we tested associations with resistance to 7 *P. horiana* isolates in the 46 cultivars (Table 4). None of the 7 isolates showed an identical infection profile on the set of 46 cultivars, indicating that each of them represents a different pathotype. TS infected the largest number of cultivars (25 out of 46). NA and TO2 also infected many cultivars, 22 and 21 cultivars, respectively. TO3 infected the smallest number of cultivars (13 out of 46). Seven isolates showed non-redundant infection interaction with at least three cultivars. A set of “Purinsesu”, “SEI17” and “Southern Shell” or a set of “Purinsesu”, “SEI17” and “Refour” can be used as differential cultivars to distinguish the seven isolates.

**Table 3:** Single nucleotide polymorphisms (SNPs) identified between SCSE_SC004884.1_65872 and SCSE_SC000716.1_75925

	Parental genotype		Corresponding SNP position on chromosome
SNPs^a^	Ariesu	Yellow Queen	9 of the *C. seticuspe* pseudomolecule (bp)
Cse2.0_LG9_46170750^T^	CCCCCT	CCCCCC	46 170 750
Cse2.0_LG9_46476925^C^	TTTTTC	TTTTTT	46 476 925
Cse2.0_LG9_47009004^G^	AAAAAG	AAAAAA	47 009 004
Cse2.0_LG9_47483611^T^	GGGGGT	GGGGGG	47 483 611
Cse2.0_LG9_48084985^C^	TTTTTC	TTTTTT	48 084 985
Cse2.0_LG9_48453417^A^	GGGGGA	GGGGGG	48 453 417
Cse2.0_LG9_49032201^A^	GGGGGA	GGGGGG	49 032 201
Cse2.0_LG9_50042598^A^	GGGGGA	GGGGGG	50 042 598

a SNPs were designed every 0.5 Mbp in the 46.1–50.0-Mbp region on the *C. seticuspe* genome sequence of chromosome 9 (CSE_r2.0, http://mum-garden.kazusa.or.jp).

**Table 4:** Resistance to seven *P. horiana* isolates and presence of resistance allele in *Phr2*-linked single nucleotide polymorphism (SNP) markers in chrysanthemum genetic resources. Cultivars that showed no telia were scored as “R”. Cultivars were scored as “S” when at least one telium was observed on the plant. *P. horiana* resistance was examined by three separate inoculations per isolate

	Resistance to *P. horiana* isolates							Presence of resistance allele in *Phr2*-linked SNP markers^a^			
Cultivar	TS	NA	AK	IB	TO1	TO2	TO3	Cse2.0_LG9_ 47009004^G^	Cse2.0_LG9_ 47483611^T^	Cse2.0_LG9_ 48084985^C^	Cse2.0_LG9_ 48453417^A^
Ariesu	R	S	R	R	R	R	R	+	+	+	+
Kyura Kids	R	R	R	R	R	R	R	−	−	−	−
Moze Cute	R	R	R	R	R	R	R	+	+	+	+
SEI03	R	R	R	R	R	R	R	+	+	+	+
SEI04	R	R	R	R	R	R	R	−	−	−	−
SEI05	R	R	R	R	R	R	R	−	−	−	−
SEI06	R	R	R	R	R	R	R	−	−	−	−
SEI07	R	R	R	R	R	R	R	−	−	−	−
SEI08	R	R	R	R	R	R	R	−	−	−	−
Southern Pegasus	R	R	R	R	R	R	R	−	−	−	−
TM Miruku	R	R	R	R	R	R	R	−	−	−	−
Kyura Shusa	R	S	R	R	R	R	R	+	+	+	+
SEI01	R	S	R	R	R	R	R	+	+	+	+
SEI02	R	S	R	R	R	R	R	+	+	+	+
Kankohbai	R	R	R	R	R	S	R	−	−	−	−
SEI09	R	R	R	R	R	S	R	−	−	−	−
SEI10	R	R	R	R	R	S	R	−	−	−	−
Sanyo-ougon	S	R	R	R	R	R	R	−	−	−	−
Seiko-no-mine	S	R	R	R	R	R	R	−	−	−	−
Westland Pink	S	R	R	R	R	R	R	−	−	−	−
SEI14	S	R	R	R	R	R	R	−	−	−	−
SEI15	S	R	R	R	R	R	R	−	−	−	−
SEI16	S	R	R	R	R	R	R	−	−	−	−
Kanseisetsu	S	R	S	R	R	R	R	−	−	−	−
Otomezakura	S	R	S	R	R	R	R	−	−	−	−
Moze Frame	R	S	R	R	R	S	R	−	−	−	−
Kanamarufuji	S	S	R	R	R	R	R	−	−	−	−
Purinsesu	R	S	R	R	S	S	R	−	−	−	−
Bunmei	S	S	R	R	S	R	R	−	−	−	−
SEI17	S	S	S	R	S	R	R	−	−	−	−
Southern Shell	R	R	S	R	S	S	S	−	−	−	−
Refour	R	R	S	S	S	S	R	−	−	−	−
Seikonohikari	S	S	R	S	S	S	R	−	−	−	−
Jimba	S	R	S	S	S	S	S	−	−	−	−
Meimon	S	S	S	S	S	S	R	−	−	−	−
Kanran	S	S	S	S	S	S	S	−	−	−	−
Kinkou	S	S	S	S	S	S	S	−	−	−	−
Penny Lane	S	S	S	S	S	S	S	−	−	−	−
SEI11	S	S	S	S	S	S	S	−	−	−	−
SEI12	S	S	S	S	S	S	S	−	−	−	−
SEI13	S	S	S	S	S	S	S	−	−	−	−
SEI18	S	S	S	S	S	S	S	−	−	−	−
Shuho-no-chikara	S	S	S	S	S	S	S	−	−	−	−
Shuho-no-kokoro	S	S	S	S	S	S	S	−	−	−	−
Snowdon	S	S	S	S	S	S	S	−	−	−	−
Yellow Queen	S	S	S	S	S	S	S	−	−	−	−

a These SNP markers co-segregated with *Phr2* in 656 F_1_ progenies derived from reciprocal crosses between “Ariesu” and “Yellow Queen”. +, presence; −, absence.

**Table 5:** Relationship between the genotype of Cse2.0_LG9_48084985^C^ and resistance to the *P. horiana* isolate TS in F_1_ populations obtained from crosses between resistant cultivars and susceptible “Yellow Queen”. Plants that showed no telia were scored as resistant. Plants were scored as susceptible when at least one telium was observed. *P. horiana* resistance was examined by three separate inoculations

		Resistance to the *P. horiana* isolate TS	
Resistant parent	Resistant C allele of Cse2.0_LG9_48084985^C^	Resistant	Susceptible
Kyura Shusa	Presence	25	0
	Absence	0	29
SEI01	Presence	17	0
	Absence	0	19
SEI02	Presence	23	0
	Absence	0	27
Moze Cute	Presence	23	0
	Absence	12	9
SEI03	Presence	37	0
	Absence	5	6

*Phr2*, developed from “Ariesu”, conferred multiple isolate-specific resistance against six isolates, TS, AK, IB, TO1, TO2 and TO3, with susceptibility to NA, as described in the section Inheritance of *P. horiana* resistance in “Ariesu”. Three cultivars, namely “Kyura Shusa”, “SEI01” and “SEI02”, showed the same interaction phenotype against isolates, and each possessed all the resistance alleles for the four SNPs associated with *Phr2*, strongly indicating that these cultivars carry *Phr2*. Ten other cultivars, “Kyura Kids”, “Moze Cute”, “SEI03”, “SEI04”, “SEI05”, “SEI06”, “SEI07”, “SEI08”, “Southern Pegasus” and “TM Miruku” showed resistance to all the tested isolates. Two of these, “Moze Cute” and “SEI03”, had all the resistance alleles for the four SNPs, indicating that *Phr2* confers resistance in these two cultivars. The interaction phenotype of complete resistance to the seven isolates indicated that “Moze Cute” and “SEI03” likely carry other resistance genes or alleles that confer resistance to the isolate NA because only *Phr2* was susceptible to NA. Ten cultivars from “Kanran” to “Snowdon” listed in Table 4 as well as “Yellow Queen” showed susceptibility to all the seven isolates.

We also investigated marker–resistance association in the progenies of five resistant cultivars carrying the resistance alleles for SNPs associated with *Phr2*: “Kyura Shusa”, “Moze Cute”, “SEI01”, “SEI02” and “SEI03”. F_1_ populations were obtained from crosses with the susceptible “Yellow Queen” and subjected to inoculation tests using the TS isolate and PCR analysis to identify the representative Cse2.0_LG9_48084985^C^ SNP marker. The close to 1:1 segregation of resistance and susceptibility in F_1_ populations derived from “Kyura Shusa”, “SEI01” and “SEI02”, along with resistance against the TS isolate, was consistent with the presence of the resistant C allele at the SNP marker Cse2.0_LG9_48084985^C^ (Table 5). The results demonstrated the marker–resistance association in these cultivars and confirmed that the SNP marker could be used for detecting resistance. Based on the proportion of resistant plants in the F_1_ populations obtained with the male parents “Moze Cute” and “SEI03”, it was concluded that these cultivars have multiple genetic loci conferring resistance against *P. horiana*. In the F_1_ population derived from “Moze Cute”, the resistance:susceptibility ratio was 35:9 (Table 5). A segregation ratio such as this is likely due to duplex × nulliplex (AAaaaa × aaaaaa) or two genetic loci of simplex × nulliplex on different homologous chromosome groups (Aaaaaa × aaaaaa and Bbbbbb × bbbbbb). Furthermore, this segregation ratio fits well into the 4:1 ratio (χ^2^ = 0.0057; *p* = 0.94) of the duplex × nulliplex compared with the 3:1 ratio (χ^2^ = 0.48; *p* = 0.47) of the two genetic loci of simplex × nulliplex; however, the population size was not sufficiently large to draw a definitive conclusion. Nevertheless, from these data, it can be estimated that “Moze Cute” has at least two resistance loci. In this population, the resistant C allele at the SNP marker Cse2.0_LG9_48084985^C^ was segregated in a ratio of 23:21 (i.e. almost 1:1), and every plant carrying the allele was resistant to the TS isolate. Thus, “Moze Cute” apparently had a single *Phr2*. In parallel, 12 F_1_ plants showed resistance without C allele amplification at the SNP marker Cse2.0_LG9_48084985^C^, indicating that an unknown genetic locus contributed to *P. horiana* resistance in these plants. Similarly, in the F_1_ population obtained from “SEI03”, the segregation ratio of 42:6 indicated the presence of multiple resistance genes or alleles in “SEI03”. At least two of those were *Phr2* because the *Phr2*-resistant allele was segregated in the ratio of almost 4:1, suggesting a duplex. Additionally, five F_1_ plants with no resistance allele exhibited resistance, indicating that “SEI03” carried another resistance gene or allele. Consequently, the results for “Moze Cute” and “SEI03” validated the SNP marker Cse2.0_LG9_48084985^C^ as a tool for distinguishing *Phr2* possession.

## Discussion

Three types of *P. horiana* resistance have been reported in chrysanthemum: complete resistance (no spore production), incomplete resistance (limited spore production) and necrosis (necrotic areas develop around the growing rust colonies and sporulation may not be completely inhibited) [8]. In this study, complete resistance was determined as “R” using a simple scoring system, in which the responses of incomplete resistance, necrosis and susceptibility were scored as “S”. Multiple cultivars with complete resistance were identified. Plants have evolved sophisticated resistance systems against pathogens and acquired many resistance genes in a gene-for-gene manner [14, 15]. In wheat, 79 resistance loci have arisen in response to *Puccinia triticina* [19, 37, 39, 43]. In barley, 26 loci conferring resistance to *Puccinia hordei* were identified [24, 36, 53, 54]. In maize, 20 genes conferring resistance to *Puccinia sorghi* were previously identified [18, 22, 41, 50]. Similarly, our results suggested that multiple resistance genes against *P. horiana* exist in chrysanthemum genetic resources [7, 8, 31, 51]. Specifically, we identified a group of resistance cultivars carrying *Phr2*. Furthermore, we found three other groups of cultivars that showed the same interaction phenotype profile against seven isolates (Table 4): (1) a group including “Kankohbai”, “SEI09” and “SEI10”; (2) a group including “Sanyo-ougon”, “Seiko-no-mine”, “Westland Pink”, “SEI14”, “SEI15” and “SEI16” and (3) a group with “Kanseisetsu” and “Otomezakura”. Cultivars in each group likely carry the same type of resistance gene. A non-redundant differential interaction phenotype was also observed in 10 cultivars: “Moze Frame”, “Kanamarufuji”, “Purinsesu”, “Bunmei”, “SEI17”, “Southern Shell”, “Refour”, “Seikonohikari”, “Jimba”, and “Meimon”. These cultivars had none of the resistance alleles for *Phr2*; therefore, it was inferred that they carry different resistance genes.

In the commercial cultivation of chrysanthemum, it is desirable to completely eliminate or control white rust. Compared with incomplete resistance and necrosis, complete resistance has the advantage of the complete absence of *P. horiana*. In addition, complete resistance shows dominant inheritance, i.e. simple Mendelian inheritance is shown even in hexaploid chrysanthemums [8, 46, 51]. In contrast, the probability of progenies inheriting incomplete resistance or necrosis is generally low [8, 51]. Thus, identifying complete resistance is advantageous for breeding resistant cultivars and achieving marker-assisted selection. Based on the present results, identifying *Phr2* resistance-conferring genes among the available chrysanthemum genetic resources is possible. So far, *Phr1* has been reported as a complete resistance locus. However, our findings (Table 4) suggest that there are other genes conferring complete resistance in chrysanthemum genetic resources. Resistance detection is also expected to become possible when a highly accurate DNA marker set is provided for analysing other genes conferring complete resistance against *P. horiana*.

Genetic analysis, DNA marker development and whole genome assembly have been difficult in chrysanthemum cultivars due to their high heterogeneity, hexaploidy and large genome size. In this study, highly accurate SNP markers were developed by fine mapping following GWAS. Previous studies have also described marker development via GWAS based on ddRAD-Seq in biparental F_1_ populations of chrysanthemum cultivars [45, 46]. Herein, we discuss fine mapping based on whole genome sequencing in chrysanthemum cultivars using the *C. seticuspe* pseudomolecule. The total lengths of the assembled genome sequences (21.0 Gbp in “Ariesu” and 20.4 Gbp in “Yellow Queen”) were longer than the previously reported estimates of, at most, 18 Gbp [32]. In contrast, the N50 lengths were relatively short, i.e. they were ultrafragmented. Such fragmentation was similarly observed in the *de novo* assemblies of diploid *Chrysanthemum* species genomes obtained at an early stage [20, 44] because repetitive sequences occupied >68.6% of the whole genome in the genus *Chrysanthemum* [20, 29, 44, 49]. In this study, the high ratio of repetitive sequences could be a major cause of the failure in assembling longer sequences in cultivated chrysanthemums. We mapped these assembled genome sequences onto the *C. seticuspe* pseudomolecule and were able to identify base variants. Indeed, we treated the *C. seticuspe* pseudomolecule as the *de facto* monoploid genome of hexaploid chrysanthemum cultivars. The resulting SNP markers were validated by fine mapping and showed transferability; therefore, the assembled genome sequences of the chrysanthemum cultivars were reliable.

The genetic order of SNP markers in the chrysanthemum linkage map was consistent with their physical positions in the *C. seticuspe* pseudomolecule, suggesting that chrysanthemum cultivars have collinearity in this genomic region with *C. seticuspe*. Collinearity between the genomes of polyploid crops and their diploid relatives has previously been reported in strawberry [10], sugarcane [48] and sweet potato [42]. Generally, robust resistance genes of cultivars are derived from ancestral donors of wild species [12, 47, 52]. A homologous gene of *Phr2* may be found in the *C. seticuspe* genome given that *C. seticuspe* is also resistant to *P. horiana* [Yamaguchi [51], who classified *C. seticuspe* as *C. boreale*]. Recently, a chromosome-level genome sequence of *C. seticuspe* was elucidated [33]. Thus, we searched the *C. seticuspe* genome for candidate genes in a 2.6-Mb genome region on chromosome 9, corresponding to a *Phr2* candidate locus in “Ariesu”. In total, 4 genes related to disease resistance (CsG_LG9.jg218308.t1.g000908.1, CsG_LG9.jg218318.t1.g000918.1, CsG_LG9.jg218352.t1.g000952.1 and CsG_LG9.jg218356.t1.g000956.1) were identified from 30 predicted genes found in the examined *C. seticuspe* region. Each gene encoded immune receptors of the nucleotide-binding site leucine-rich repeat (NBS-LRR) protein; this gene class is involved in disease resistance in plants [11]. However, we could not search for a *Phr2* locus in “Ariesu” with such NBS-LRR genes because the genome sequence is fragmented and discontinuous. In future studies, the collinearity may be used for the positional cloning of causal genes in chrysanthemum cultivars.

In conclusion, this study identified a new resistance locus and DNA markers for resistance against white rust caused by *P. horiana* in chrysanthemum cultivars using ddRAD-Seq analysis and assembled genome sequence comparison with wild *C. seticuspe* pseudomolecule as a *de facto* monoploid genome. Notably, although the assembled genome sequence of the chrysanthemum cultivar was severely fragmented, it could be reconstructed with satisfactory quality for DNA marker development by mapping onto the *C. seticuspe* pseudomolecule. Our results demonstrated this method’s utility in providing a model for DNA marker development in chrysanthemum cultivars.

## Materials and methods

### Plant materials

We prepared 46 cultivars, which were selected based on previous reports [8, 23, 31, 38, 46, 51] and anecdotal reports of infections from breeders. The details of these cultivars are provided in Supplementary data Table S1.

A pair of F_1_ populations originating from reciprocal crosses between spray-type “Ariesu” and spray-type “Yellow Queen” was produced. In total, 64 seedlings from “Ariesu” × “Yellow Queen” and 219 seedlings from “Yellow Queen” × “Ariesu” were obtained for ddRAD-Seq and genome-wide association study (GWAS). Another F_1_ population (n = 373) additionally obtained from “Yellow Queen” × “Ariesu” was used for fine-mapping analysis. Using several resistance spray-type cultivars (“Kyura Shusa”, “Moze Cute”, “SEI01”, “SEI02” and “SEI03”) as pollen parents, crosses with “Yellow Queen” were made, resulting in 54, 44, 36, 50 and 48 F_1_ seedlings, respectively, for the analysis of single nucleotide polymorphism (SNP) marker–resistance association.

Stock plants were planted in plastic pots (internal diameter, 12 cm; one stock plant for each genotype per pot) containing horticultural soil (Yokabaido, Hokkaido Peatmoss Co., Ltd.) and maintained in the vegetative state under 6-h night-break conditions (natural photoperiod with a night-break from 2200 to 0400 h using fluorescent white-light lamps) in a glasshouse at 18°C–25°C. Cuttings for the inoculation assay and samples for DNA extraction were obtained from the stock plants.

### Fungal isolates

Seven *P. horiana* isolates (codes: TS, NA, AK, IB, TO1, TO2 and TO3) were collected from cut flower growers or agricultural experimental stations in Japan in 2018 (Supplementary data Table S2). *P. horiana* is an autoecious fungus; thus, each isolate was maintained on *P. horiana*-free plants (susceptible “Shuho-no-chikara”) as described by Alaei *et al*. [1] and De Backer *et al*. [7]. The plants were grown in growth chambers maintained at 20°C and 70% relative humidity (RH) with a 16-h photoperiod provided using fluorescent white-light tubes.

### Inoculation method

Inoculation was performed in a polystyrene foam box as described by Sumitomo *et al.* [46]. Briefly, chrysanthemum rooted cuttings were put at the bottom of the box. The top opening of the box was covered with a 5-mm mesh plastic net. The heavily infected leaves were placed on the net with the telia pointing downwards. Demineralised water was used to mist the cuttings, inoculum and inside of the box. After the box was closed, it was placed in a growth chamber under dark conditions at 19°C. Subsequently, 16 h after inoculation, the rooted cuttings were transferred into a growth chamber (22°C, 70% RH, 16-h photoperiod). Symptoms were evaluated 35 days after inoculation.

Inoculation assays were performed to investigate the susceptibility of the 46 cultivars to each of the 7 *P. horiana* isolates (TS, NA, AK, IB, TO1, TO2 and TO3). Isolate TS was used for F_1_ populations originating from crosses between “Yellow Queen” and cultivars “Ariesu”, “Kyura Shusa”, “Moze Cute”, “SEI01”, “SEI02” and “SEI03”. One rooted cutting per genotype was used in one assay, and the assay was repeated three times per isolate in different experiments.

### Phenotyping

A simple scoring system, susceptible (S) or resistant (R), was used to identify complete resistance, which is the strongest of the three *P. horiana* resistance types [8]. If at least one telium was observed on a plant in any of the assays, the phenotype was scored as “S”, whereas plants that showed no telia at all were scored as “R”.

### d‌dRAD-Seq analysis

F_1_ populations originating from “Ariesu” × “Yellow Queen” (n = 64) and “Yellow Queen” × “Ariesu” (n = 219) and the parents (“Ariesu” and “Yellow Queen”) were used. Genomic DNA was isolated from the shoot tips (fresh weight, 30 mg) using a DNeasy Plant Mini Kit according to the manufacturer’s instructions (Qiagen, Hilden, Germany). ddRAD-Seq libraries were prepared as described by Sumitomo *et al*. [45] and sequenced using HiSeq 2000 and MiSeq platforms (Illumina). The sequence reads were registered in the Sequence Read Archive database in the DNA Data Bank of Japan (accession numbers DRA007925, DRA011746 and DRA011899).

### Data processing and simplex SNP mining

Data processing of sequence reads and simplex SNP calling were performed as described by Sumitomo *et al*. [45]. Briefly, high-quality ddRAD-Seq reads were mapped onto the reference *C. seticuspe* genome sequence (CSE_r1.0; [20]) using Bowtie 2 (version 2.2.3; [26]) with parameters of maximum fragment size length, 1000 (I = 1000), in the “—sensitive” preset of the “—end-to-end” mode. The resultant sequence alignment/map format files were converted to binary sequence alignment/map format files and subjected to SNP calling using the mpileup option of SAMtools (version 0.1.19; [28]) and the mpileup2snp option of VarScan 2 (version 2.3; [25]) to obtain a variant call format file that included SNP information. High-confidence SNPs were called from the resulting sequence alignments using the following criteria in VCFtools (version 0.1.12b; [6]): (i) depth of coverage: ≥10 for each SNP position in each F_1_ individual; (ii) proportion of F_1_ individuals with missing data in F_1_ population: <0.25 for each locus.

The number of reads in the pooled F_1_ progeny samples was used to estimate the genotype of the parental cultivars at each SNP locus [2, 42]. Simplex and double-simplex SNPs were selected according to the alternative allele frequency (AAF) of the pooled F_1_ progeny samples. The AAF for each position was computed by dividing the number of reads with the bases supporting a variant by the number of total reads aligned at the position. For example, the AAF of simplex SNP sites (AAAAA × AAAAAa) was 0.0833 (1/12). Accordingly, SNPs with 0.0417 ≤ AAF < 0.1250 were selected as simplex SNPs. Each individual’s genotype was determined for the simplex and double-simplex SNP loci. AAFs equal to 0 or > 0.0000 were scored as homozygous or non-homozygous reference alleles, respectively, in each F_1_ individual. In addition, segregation data subsets from simplex loci that fitted the expected ratio of 1:1 were selected based on the function of chisq.test() of R software (*P* > 0.01). For more information, refer to Sumitomo *et al*. [45].

### GWAS

For a broadly diverse population, typical GWAS is performed. However, we used biparental populations for GWAS, wherein the SNP allele frequency could be predicted. The association study was performed in F_1_ populations consisting 64 seedlings from “Ariesu” × “Yellow Queen” and 219 seedlings from “Yellow Queen” × “Ariesu” using the TASSEL programme (with the default parameters) to run a general linear model [5]. Thresholds for the association were set at 4.6 × 10^−6^ (= 0.05/10779) at a 5% significance level after implementing a Bonferroni multiple test correction [4].

### Linkage analysis

Linkage analysis was performed using JoinMap v4.1 software (Kyazma B.V., Wageningen, The Netherlands) based on the backcross (BC1) option with a limit of determination threshold of 10.0. The genetic distance between markers in a map was calculated using regression mapping and the Kosambi mapping function.

### Fine mapping by comparing “Ariesu” and “Yellow Queen” whole genome sequences

The genomic DNA of “Ariesu” and “Yellow Queen” was used to construct paired-end libraries. The expected insert size of the paired-end libraries was 500 bp, and the sequences were generated using Illumina HiSeqX (Illumina, San Diego, CA, USA) with a 151-nt read length. The generated reads were assembled using SOAPdenovo2 with a k-mer of 101 [30]. Assembled sequences that were > 500 bp in size were mapped onto the *C. seticuspe* pseudomolecule (CSE_r2.0; http://mum-garden.kazusa.or.jp) using minimap2 software [27].

We used IGV software [40] to browse and compare the resultant sequence alignments and identified base variants between ddRAD-Seq markers, SCSE_SC004884.1_65872 and SCSE_SC000716.1_75925. The corresponding positions of these markers were 44 717 622 and 52 150 792 bp, respectively, on the *C. seticuspe* chromosome 9. Therefore, we identified SNPs from the assembled genome sequences of “Ariesu” and “Yellow Queen” mapped onto the 44 717 622 − 52 150 792-bp region of the *C. seticuspe* chromosome 9. SNPs determined as heterozygous and homozygous were identified in “Ariesu” and “Yellow Queen”, respectively, as SNP marker candidates. The markers showing linkage to the minor C allele on CSE_SC004884.1_65872 and minor G allele on SCSE_SC000716.1_75925 were screened by polymerase chain reaction (PCR) analysis using DNA from “Ariesu”, “Yellow Queen” and the two bulks of resistant or susceptible F_1_ plants. Information related to the primers used is shown in Supplementary data Table S3. SNP-distinguishable PCR was performed as described by Sumitomo *et al.* [46].

### Analysis of SNP marker–resistance association

SNP marker–resistance association was investigated in the genetic resources. For 46 cultivars, the presence or absence of the resistance-linked allele was investigated at four SNP positions (Cse2.0_LG9_47009004^G^, Cse2.0_LG9_47483611^T^, Cse2.0_LG9_48084985^C^ and Cse2.0_LG9_48453417^A^) co-segregated with *Phr2* resistance in F_1_ progenies derived from “Ariesu”. Marker–resistance association was also investigated in progenies originating from five resistant cultivars: “Kyura Shusa”, “Moze Cute”, “SEI01”, “SEI02” and “SEI03”. The representative Cse2.0_LG9_48084985^C^ SNP marker was used for PCR analysis. The detailed information on the plant material and evaluation of *P. horiana* resistance have been described in the “Plant materials” and “Inoculation methods” sections, respectively. For these analyses, genomic DNA was extracted from the expanding leaves (fresh weight, 20 mg) using DNAs-ici!-P (Rizo Inc., Tsukuba, Japan). SNP-distinguishable PCR was performed as described above using 1 μl of DNA solution.

## Acknowledgments

This work was partially supported by funds from the Kazusa DNA Research Institute Foundation. We thank Inochio Seikoen, National Federation of Agricultural Cooperative Associations, Genebank Project (NARO) and Kagoshima Prefectural Institute for Agricultural Development for supplying the cuttings. We thank A. Yamagata, S. Murazaki, Y. Shima and S. Asano for supplying *P. horiana* isolates. We also thank S. Kamei and Y. Hirakawa (NARO) as well as S. Sasamoto, S. Nakayama, H. Tsuruoka, C. Minami, A. Watanabe and Y. Kishida (Kazusa DNA Research Institute) for their technical assistance. Lastly, we thank Enago (www.enago.jp) for the English language review.

## Author contributions

KSu designed and executed the study, prepared all tables and figures and wrote the manuscript. KSh assisted in ddRAD-Seq analysis and GWAS. HH, KSh and SI conducted comparative genome analysis. AH conducted part of the inoculation test. MN, YN, TH, MK, HY and FT contributed to data analysis and redrafting of the manuscript.

## Availability of data and materials

All phenotypic and genotypic data are provided as Supplementary Material.

## Conflicts of interest

The authors declare that they have no competing interests.

## Supplementary data

Supplementary data is available at *Horticulture Research * online.

## Supplementary Material

Web_Material_uhac170
