# Supplementary material for: A genome-wide association and fine-mapping study of white rust resistance in hexaploid chrysanthemum cultivars with a wild diploid reference genome
Source: Hortic Res. 2022 Aug 3;9:uhac170. doi: 10.1093/hr/uhac170 (PMC9613985; doi:10.1093/hr/uhac170)
Supplement: Web_Material_uhac170 [file web_material_uhac170.zip › SupplementaryFigS1_r1.pptx]

## Slide 1
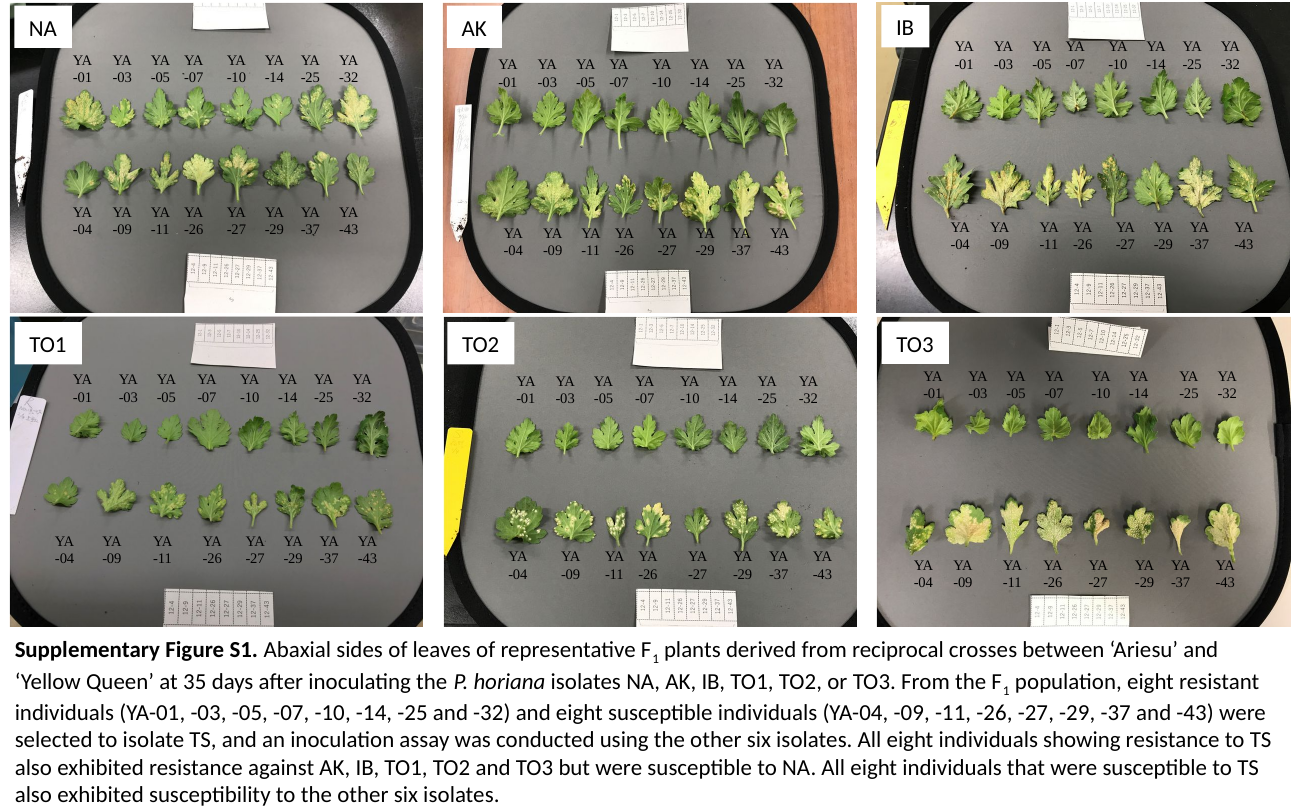

IB
NA
AK
YA
-01
YA
-03
YA
-05
YA
-07
YA
-10
YA
-14
YA
-25
YA
-32
YA
-01
YA
-03
YA
-05
YA
-07
YA
-10
YA
-14
YA
-25
YA
-32
YA
-01
YA
-03
YA
-05
YA
-07
YA
-10
YA
-14
YA
-25
YA
-32
YA
-04
YA
-09
YA
-11
YA
-26
YA
-27
YA
-29
YA
-37
YA
-43
YA
-04
YA
-09
YA
-11
YA
-26
YA
-27
YA
-29
YA
-37
YA
-43
YA
-04
YA
-09
YA
-11
YA
-26
YA
-27
YA
-29
YA
-37
YA
-43
TO3
TO2
TO1
YA
-01
YA
-03
YA
-05
YA
-07
YA
-10
YA
-14
YA
-25
YA
-32
YA
-01
YA
-03
YA
-05
YA
-07
YA
-10
YA
-14
YA
-25
YA
-32
YA
-01
YA
-03
YA
-05
YA
-07
YA
-10
YA
-14
YA
-25
YA
-32
YA
-04
YA
-09
YA
-11
YA
-26
YA
-27
YA
-29
YA
-37
YA
-43
YA
-04
YA
-09
YA
-11
YA
-26
YA
-27
YA
-29
YA
-37
YA
-43
YA
-04
YA
-09
YA
-11
YA
-26
YA
-27
YA
-29
YA
-37
YA
-43
Supplementary Figure S1. Abaxial sides of leaves of representative F1 plants derived from reciprocal crosses between ‘Ariesu’ and ‘Yellow Queen’ at 35 days after inoculating the P. horiana isolates NA, AK, IB, TO1, TO2, or TO3. From the F1 population, eight resistant individuals (YA-01, -03, -05, -07, -10, -14, -25 and -32) and eight susceptible individuals (YA-04, -09, -11, -26, -27, -29, -37 and -43) were selected to isolate TS, and an inoculation assay was conducted using the other six isolates. All eight individuals showing resistance to TS also exhibited resistance against AK, IB, TO1, TO2 and TO3 but were susceptible to NA. All eight individuals that were susceptible to TS also exhibited susceptibility to the other six isolates.
