# Supplementary material for: A genome-wide association and fine-mapping study of white rust resistance in hexaploid chrysanthemum cultivars with a wild diploid reference genome
Source: Hortic Res. 2022 Aug 3;9:uhac170. doi: 10.1093/hr/uhac170 (PMC9613985; doi:10.1093/hr/uhac170)
Supplement: Web_Material_uhac170 [file web_material_uhac170.zip › PTandGT_r1.doc]

| Table. Marker genotypes and *P. horiana* resistance in 283 F_1_ plants obtained from reciprocal crosses between ‘Ariesu’ and ‘Yellow Queen’ | | | |  |  |  |  |  |  |  |  |
| --- | --- | --- | --- | --- | --- | --- | --- | --- | --- | --- | --- |
| Sample Name | Resistance to *P. horiana*^a^ | SCSE_SC004884.1_65872^b^ | SCSE_SC000716.1_75925 |  |  |  |  |  |  |  |  |
| YA-01 | R | TTTTTC | AAAAAG |  |  |  |  |  |  |  |  |
| YA-02 | S | TTTTTT | AAAAAA |  |  |  |  |  |  |  |  |
| YA-03 | R | TTTTTC | AAAAAG |  |  |  |  |  |  |  |  |
| YA-04 | S | TTTTTT | AAAAAA |  |  |  |  |  |  |  |  |
| YA-05 | R | TTTTTC | AAAAAG |  |  |  |  |  |  |  |  |
| YA-06 | R | TTTTTC | AAAAAG |  |  |  |  |  |  |  |  |
| YA-07 | R | TTTTTC | AAAAAG |  |  |  |  |  |  |  |  |
| YA-08 | R | TTTTTC | AAAAAG |  |  |  |  |  |  |  |  |
| YA-09 | S | TTTTTT | AAAAAA |  |  |  |  |  |  |  |  |
| YA-10 | R | TTTTTC | AAAAAG |  |  |  |  |  |  |  |  |
| YA-11 | S | TTTTTT | AAAAAA |  |  |  |  |  |  |  |  |
| YA-12 | S | TTTTTT | AAAAAA |  |  |  |  |  |  |  |  |
| YA-13 | S | TTTTTT | AAAAAA |  |  |  |  |  |  |  |  |
| YA-14 | R | TTTTTC | AAAAAG |  |  |  |  |  |  |  |  |
| YA-15 | S | TTTTTT | AAAAAA |  |  |  |  |  |  |  |  |
| YA-16 | S | TTTTTT | AAAAAA |  |  |  |  |  |  |  |  |
| YA-17 | R | TTTTTC | AAAAAG |  |  |  |  |  |  |  |  |
| YA-18 | R | TTTTTC | AAAAAG |  |  |  |  |  |  |  |  |
| YA-19 | S | TTTTTT | AAAAAA |  |  |  |  |  |  |  |  |
| YA-20 | S | TTTTTT | AAAAAA |  |  |  |  |  |  |  |  |
| YA-21 | S | TTTTTT | AAAAAA |  |  |  |  |  |  |  |  |
| YA-22 | R | TTTTTC | AAAAAG |  |  |  |  |  |  |  |  |
| YA-23 | S | TTTTTT | AAAAAA |  |  |  |  |  |  |  |  |
| YA-24 | S | TTTTTT | AAAAAA |  |  |  |  |  |  |  |  |
| YA-25 | R | TTTTTT | AAAAAG |  |  |  |  |  |  |  |  |
| YA-26 | S | TTTTTT | AAAAAA |  |  |  |  |  |  |  |  |
| YA-27 | S | TTTTTT | AAAAAA |  |  |  |  |  |  |  |  |
| YA-28 | R | TTTTTC | AAAAAG |  |  |  |  |  |  |  |  |
| YA-29 | S | TTTTTT | AAAAAA |  |  |  |  |  |  |  |  |
| YA-30 | R | TTTTTC | AAAAAG |  |  |  |  |  |  |  |  |
| YA-31 | S | TTTTTT | AAAAAA |  |  |  |  |  |  |  |  |
| YA-32 | R | TTTTTC | AAAAAG |  |  |  |  |  |  |  |  |
| YA-33 | S | TTTTTT | AAAAAA |  |  |  |  |  |  |  |  |
| YA-34 | S | TTTTTT | AAAAAA |  |  |  |  |  |  |  |  |
| YA-36 | R | TTTTTC | AAAAAG |  |  |  |  |  |  |  |  |
| YA-37 | S | TTTTTT | AAAAAA |  |  |  |  |  |  |  |  |
| YA-38 | S | TTTTTT | AAAAAA |  |  |  |  |  |  |  |  |
| YA-39 | R | TTTTTC | AAAAAG |  |  |  |  |  |  |  |  |
| YA-40 | R | TTTTTC | AAAAAG |  |  |  |  |  |  |  |  |
| YA-41 | S | TTTTTT | AAAAAA |  |  |  |  |  |  |  |  |
| YA-42 | R | TTTTTC | AAAAAG |  |  |  |  |  |  |  |  |
| YA-43 | S | TTTTTT | AAAAAA |  |  |  |  |  |  |  |  |
| YA-44 | S | TTTTTT | AAAAAA |  |  |  |  |  |  |  |  |
| YA-45 | R | TTTTTC | AAAAAG |  |  |  |  |  |  |  |  |
| YA-46 | R | TTTTTC | AAAAAG |  |  |  |  |  |  |  |  |
| YA-47 | R | TTTTTC | AAAAAG |  |  |  |  |  |  |  |  |
| YA-49 | S | TTTTTT | AAAAAA |  |  |  |  |  |  |  |  |
| YA-50 | S | TTTTTT | AAAAAG |  |  |  |  |  |  |  |  |
| YA-51 | R | TTTTTC | AAAAAG |  |  |  |  |  |  |  |  |
| YA-52 | R | TTTTTC | AAAAAG |  |  |  |  |  |  |  |  |
| AY-01 | S | TTTTTT | AAAAAA |  |  |  |  |  |  |  |  |
| AY-02 | R | TTTTTC | AAAAAG |  |  |  |  |  |  |  |  |
| AY-03 | R | TTTTTC | AAAAAG |  |  |  |  |  |  |  |  |
| AY-04 | S | TTTTTT | AAAAAA |  |  |  |  |  |  |  |  |
| AY-05 | S | TTTTTT | AAAAAA |  |  |  |  |  |  |  |  |
| AY-06 | R | TTTTTC | AAAAAG |  |  |  |  |  |  |  |  |
| AY-07 | R | TTTTTC | AAAAAG |  |  |  |  |  |  |  |  |
| AY-09 | R | TTTTTC | AAAAAG |  |  |  |  |  |  |  |  |
| AY-10 | R | TTTTTC | AAAAAG |  |  |  |  |  |  |  |  |
| AY-11 | S | TTTTTT | AAAAAA |  |  |  |  |  |  |  |  |
| AY-12 | R | TTTTTC | AAAAAG |  |  |  |  |  |  |  |  |
| AY-13 | R | TTTTTC | AAAAAG |  |  |  |  |  |  |  |  |
| AY-14 | R | TTTTTC | AAAAAG |  |  |  |  |  |  |  |  |
| AY-15 | R | TTTTTC | AAAAAG |  |  |  |  |  |  |  |  |
| AY-16 | R | TTTTTC | AAAAAG |  |  |  |  |  |  |  |  |
| AY-17 | R | TTTTTC | AAAAAG |  |  |  |  |  |  |  |  |
| AY-18 | S | TTTTTT | AAAAAA |  |  |  |  |  |  |  |  |
| AY-19 | R | TTTTTC | AAAAAG |  |  |  |  |  |  |  |  |
| AY-20 | S | TTTTTT | AAAAAA |  |  |  |  |  |  |  |  |
| AY-21 | S | TTTTTT | AAAAAA |  |  |  |  |  |  |  |  |
| AY-22 | R | TTTTTC | AAAAAG |  |  |  |  |  |  |  |  |
| AY-23 | R | TTTTTC | AAAAAG |  |  |  |  |  |  |  |  |
| AY-24 | R | TTTTTC | AAAAAG |  |  |  |  |  |  |  |  |
| AY-25 | R | TTTTTC | AAAAAG |  |  |  |  |  |  |  |  |
| AY-26 | R | TTTTTC | AAAAAG |  |  |  |  |  |  |  |  |
| AY-27 | R | TTTTTC | AAAAAG |  |  |  |  |  |  |  |  |
| AY-28 | S | TTTTTT | AAAAAA |  |  |  |  |  |  |  |  |
| AY-29 | R | TTTTTC | AAAAAG |  |  |  |  |  |  |  |  |
| AY-30 | R | TTTTTC | AAAAAG |  |  |  |  |  |  |  |  |
| AY-31 | S | TTTTTT | AAAAAA |  |  |  |  |  |  |  |  |
| AY-32 | R | TTTTTC | AAAAAG |  |  |  |  |  |  |  |  |
| AY-33 | S | TTTTTT | AAAAAA |  |  |  |  |  |  |  |  |
| AY-34 | R | TTTTTC | AAAAAG |  |  |  |  |  |  |  |  |
| AY-35 | S | TTTTTT | AAAAAA |  |  |  |  |  |  |  |  |
| AY-36 | S | TTTTTT | AAAAAA |  |  |  |  |  |  |  |  |
| AY-37 | S | TTTTTT | AAAAAA |  |  |  |  |  |  |  |  |
| AY-38 | R | TTTTTC | AAAAAG |  |  |  |  |  |  |  |  |
| AY-39 | R | TTTTTC | AAAAAG |  |  |  |  |  |  |  |  |
| AY-40 | S | TTTTTT | AAAAAA |  |  |  |  |  |  |  |  |
| AY-41 | S | TTTTTT | AAAAAA |  |  |  |  |  |  |  |  |
| AY-42 | S | TTTTTT | AAAAAA |  |  |  |  |  |  |  |  |
| AY-43 | S | TTTTTT | AAAAAA |  |  |  |  |  |  |  |  |
| AY-44 | S | TTTTTT | AAAAAA |  |  |  |  |  |  |  |  |
| AY-45 | S | TTTTTT | AAAAAA |  |  |  |  |  |  |  |  |
| AY-46 | R | TTTTTC | AAAAAG |  |  |  |  |  |  |  |  |
| AY-47 | S | TTTTTT | AAAAAA |  |  |  |  |  |  |  |  |
| AY-48 | R | TTTTTC | AAAAAG |  |  |  |  |  |  |  |  |
| AY-49 | S | TTTTTC | AAAAAA |  |  |  |  |  |  |  |  |
| AY-50 | R | TTTTTC | AAAAAG |  |  |  |  |  |  |  |  |
| AY-51 | S | TTTTTT | AAAAAA |  |  |  |  |  |  |  |  |
| AY-52 | S | TTTTTT | AAAAAA |  |  |  |  |  |  |  |  |
| AY-53 | S | TTTTTT | AAAAAA |  |  |  |  |  |  |  |  |
| AY-54 | R | TTTTTC | AAAAAG |  |  |  |  |  |  |  |  |
| AY-55 | S | TTTTTT | AAAAAA |  |  |  |  |  |  |  |  |
| AY-56 | R | TTTTTC | AAAAAG |  |  |  |  |  |  |  |  |
| AY-57 | S | TTTTTT | AAAAAA |  |  |  |  |  |  |  |  |
| AY-58 | R | TTTTTC | AAAAAG |  |  |  |  |  |  |  |  |
| AY-59 | R | TTTTTC | AAAAAG |  |  |  |  |  |  |  |  |
| AY-60 | R | TTTTTC | AAAAAG |  |  |  |  |  |  |  |  |
| AY-61 | S | TTTTTT | AAAAAA |  |  |  |  |  |  |  |  |
| AY-62 | S | TTTTTT | AAAAAA |  |  |  |  |  |  |  |  |
| AY-63 | S | TTTTTT | AAAAAA |  |  |  |  |  |  |  |  |
| AY-64 | S | TTTTTT | AAAAAA |  |  |  |  |  |  |  |  |
| AY-65 | R | TTTTTC | AAAAAG |  |  |  |  |  |  |  |  |
| YA 1 | R | TTTTTC | AAAAAG |  |  |  |  |  |  |  |  |
| YA 2 | R | TTTTTC | AAAAAG |  |  |  |  |  |  |  |  |
| YA 3 | R | TTTTTC | AAAAAG |  |  |  |  |  |  |  |  |
| YA 4 | R | TTTTTC | AAAAAG |  |  |  |  |  |  |  |  |
| YA 5 | S | TTTTTT | AAAAAA |  |  |  |  |  |  |  |  |
| YA 6 | S | TTTTTT | AAAAAA |  |  |  |  |  |  |  |  |
| YA 7 | R | TTTTTC | AAAAAG |  |  |  |  |  |  |  |  |
| YA 8 | R | TTTTTC | AAAAAG |  |  |  |  |  |  |  |  |
| YA 9 | S | TTTTTT | AAAAAA |  |  |  |  |  |  |  |  |
| YA 10 | R | TTTTTC | AAAAAG |  |  |  |  |  |  |  |  |
| YA 11 | S | TTTTTT | AAAAAA |  |  |  |  |  |  |  |  |
| YA 12 | S | TTTTTT | AAAAAA |  |  |  |  |  |  |  |  |
| YA 13 | S | TTTTTT | AAAAAG |  |  |  |  |  |  |  |  |
| YA 14 | R | TTTTTC | AAAAAG |  |  |  |  |  |  |  |  |
| YA 15 | R | TTTTTC | AAAAAG |  |  |  |  |  |  |  |  |
| YA 16 | R | TTTTTC | AAAAAG |  |  |  |  |  |  |  |  |
| YA 17 | R | TTTTTC | AAAAAG |  |  |  |  |  |  |  |  |
| YA 18 | S | TTTTTT | AAAAAA |  |  |  |  |  |  |  |  |
| YA 19 | S | TTTTTT | AAAAAA |  |  |  |  |  |  |  |  |
| YA 20 | S | TTTTTT | AAAAAA |  |  |  |  |  |  |  |  |
| YA 21 | R | TTTTTC | AAAAAG |  |  |  |  |  |  |  |  |
| YA 22 | R | TTTTTC | AAAAAG |  |  |  |  |  |  |  |  |
| YA 23 | R | TTTTTC | AAAAAG |  |  |  |  |  |  |  |  |
| YA 24 | S | TTTTTT | AAAAAA |  |  |  |  |  |  |  |  |
| YA 25 | R | TTTTTC | AAAAAG |  |  |  |  |  |  |  |  |
| YA 26 | R | TTTTTC | AAAAAG |  |  |  |  |  |  |  |  |
| YA 27 | S | TTTTTT | AAAAAA |  |  |  |  |  |  |  |  |
| YA 28 | S | TTTTTT | AAAAAA |  |  |  |  |  |  |  |  |
| YA 29 | S | TTTTTT | AAAAAA |  |  |  |  |  |  |  |  |
| YA 30 | R | TTTTTC | AAAAAG |  |  |  |  |  |  |  |  |
| YA 31 | S | TTTTTT | AAAAAA |  |  |  |  |  |  |  |  |
| YA 32 | S | TTTTTT | AAAAAA |  |  |  |  |  |  |  |  |
| YA 33 | R | TTTTTC | AAAAAG |  |  |  |  |  |  |  |  |
| YA 34 | S | TTTTTT | AAAAAA |  |  |  |  |  |  |  |  |
| YA 35 | R | TTTTTC | AAAAAG |  |  |  |  |  |  |  |  |
| YA 36 | S | TTTTTT | AAAAAA |  |  |  |  |  |  |  |  |
| YA 37 | R | TTTTTC | AAAAAG |  |  |  |  |  |  |  |  |
| YA 38 | R | TTTTTC | AAAAAG |  |  |  |  |  |  |  |  |
| YA 39 | R | TTTTTC | AAAAAG |  |  |  |  |  |  |  |  |
| YA 40 | S | TTTTTT | AAAAAA |  |  |  |  |  |  |  |  |
| YA 41 | S | TTTTTT | AAAAAA |  |  |  |  |  |  |  |  |
| YA 42 | R | TTTTTC | AAAAAG |  |  |  |  |  |  |  |  |
| YA 43 | R | TTTTTC | AAAAAG |  |  |  |  |  |  |  |  |
| YA 44 | R | TTTTTC | AAAAAG |  |  |  |  |  |  |  |  |
| YA 45 | S | TTTTTT | AAAAAA |  |  |  |  |  |  |  |  |
| YA 46 | S | TTTTTT | AAAAAA |  |  |  |  |  |  |  |  |
| YA 47 | R | TTTTTC | AAAAAG |  |  |  |  |  |  |  |  |
| YA 48 | S | TTTTTT | AAAAAA |  |  |  |  |  |  |  |  |
| YA 49 | S | TTTTTT | AAAAAG |  |  |  |  |  |  |  |  |
| YA 50 | S | TTTTTT | AAAAAA |  |  |  |  |  |  |  |  |
| YA 51 | R | TTTTTC | AAAAAG |  |  |  |  |  |  |  |  |
| YA 52 | R | TTTTTC | AAAAAG |  |  |  |  |  |  |  |  |
| YA 53 | R | TTTTTC | AAAAAG |  |  |  |  |  |  |  |  |
| YA 54 | S | TTTTTT | AAAAAA |  |  |  |  |  |  |  |  |
| YA 55 | R | TTTTTC | AAAAAG |  |  |  |  |  |  |  |  |
| YA 56 | R | TTTTTC | AAAAAG |  |  |  |  |  |  |  |  |
| YA 57 | R | TTTTTC | AAAAAG |  |  |  |  |  |  |  |  |
| YA 58 | R | TTTTTC | AAAAAG |  |  |  |  |  |  |  |  |
| YA 59 | S | TTTTTT | AAAAAA |  |  |  |  |  |  |  |  |
| YA 60 | R | TTTTTC | AAAAAG |  |  |  |  |  |  |  |  |
| YA 61 | S | TTTTTT | AAAAAA |  |  |  |  |  |  |  |  |
| YA 62 | S | TTTTTT | AAAAAA |  |  |  |  |  |  |  |  |
| YA 63 | R | TTTTTC | AAAAAG |  |  |  |  |  |  |  |  |
| YA 64 | S | TTTTTT | AAAAAA |  |  |  |  |  |  |  |  |
| YA 65 | R | TTTTTC | AAAAAG |  |  |  |  |  |  |  |  |
| YA 66 | R | TTTTTC | AAAAAG |  |  |  |  |  |  |  |  |
| YA 67 | S | TTTTTT | AAAAAA |  |  |  |  |  |  |  |  |
| YA 68 | S | TTTTTT | AAAAAA |  |  |  |  |  |  |  |  |
| YA 69 | S | TTTTTT | AAAAAA |  |  |  |  |  |  |  |  |
| YA 70 | S | TTTTTT | AAAAAA |  |  |  |  |  |  |  |  |
| YA 71 | R | TTTTTC | AAAAAG |  |  |  |  |  |  |  |  |
| YA 72 | S | TTTTTT | AAAAAA |  |  |  |  |  |  |  |  |
| YA 73 | S | TTTTTT | AAAAAA |  |  |  |  |  |  |  |  |
| YA 74 | S | TTTTTT | AAAAAA |  |  |  |  |  |  |  |  |
| YA 75 | R | TTTTTC | AAAAAG |  |  |  |  |  |  |  |  |
| YA 76 | S | TTTTTT | AAAAAA |  |  |  |  |  |  |  |  |
| YA 77 | R | TTTTTC | AAAAAG |  |  |  |  |  |  |  |  |
| YA 78 | R | TTTTTC | AAAAAG |  |  |  |  |  |  |  |  |
| YA 79 | R | TTTTTC | AAAAAG |  |  |  |  |  |  |  |  |
| YA 80 | S | TTTTTT | AAAAAA |  |  |  |  |  |  |  |  |
| YA 81 | R | TTTTTC | AAAAAG |  |  |  |  |  |  |  |  |
| YA 82 | S | TTTTTT | AAAAAA |  |  |  |  |  |  |  |  |
| YA 83 | S | TTTTTT | AAAAAA |  |  |  |  |  |  |  |  |
| YA 84 | R | TTTTTC | AAAAAG |  |  |  |  |  |  |  |  |
| YA 85 | S | TTTTTT | AAAAAA |  |  |  |  |  |  |  |  |
| YA 86 | S | TTTTTT | AAAAAA |  |  |  |  |  |  |  |  |
| YA 87 | R | TTTTTT | AAAAAG |  |  |  |  |  |  |  |  |
| YA 88 | R | TTTTTC | AAAAAG |  |  |  |  |  |  |  |  |
| YA 89 | R | TTTTTC | AAAAAG |  |  |  |  |  |  |  |  |
| YA 90 | R | TTTTTC | AAAAAG |  |  |  |  |  |  |  |  |
| YA 91 | R | TTTTTC | AAAAAG |  |  |  |  |  |  |  |  |
| YA 92 | S | TTTTTT | AAAAAA |  |  |  |  |  |  |  |  |
| YA 93 | S | TTTTTT | AAAAAA |  |  |  |  |  |  |  |  |
| YA 94 | S | TTTTTT | AAAAAA |  |  |  |  |  |  |  |  |
| YA 95 | R | TTTTTC | AAAAAG |  |  |  |  |  |  |  |  |
| YA 96 | R | TTTTTC | AAAAAG |  |  |  |  |  |  |  |  |
| YA 97 | S | TTTTTT | AAAAAA |  |  |  |  |  |  |  |  |
| YA 98 | R | TTTTTC | AAAAAG |  |  |  |  |  |  |  |  |
| YA 99 | S | TTTTTT | AAAAAA |  |  |  |  |  |  |  |  |
| YA 100 | R | TTTTTC | AAAAAG |  |  |  |  |  |  |  |  |
| YA 101 | S | TTTTTT | AAAAAA |  |  |  |  |  |  |  |  |
| YA 102 | R | TTTTTC | AAAAAG |  |  |  |  |  |  |  |  |
| YA 103 | S | TTTTTT | AAAAAA |  |  |  |  |  |  |  |  |
| YA 104 | R | TTTTTC | AAAAAG |  |  |  |  |  |  |  |  |
| YA 105 | R | TTTTTC | AAAAAG |  |  |  |  |  |  |  |  |
| YA 106 | R | TTTTTC | AAAAAG |  |  |  |  |  |  |  |  |
| YA 107 | R | TTTTTC | AAAAAG |  |  |  |  |  |  |  |  |
| YA 108 | R | TTTTTC | AAAAAG |  |  |  |  |  |  |  |  |
| YA 109 | R | TTTTTC | AAAAAG |  |  |  |  |  |  |  |  |
| YA 110 | S | TTTTTT | AAAAAA |  |  |  |  |  |  |  |  |
| YA 111 | S | TTTTTT | AAAAAA |  |  |  |  |  |  |  |  |
| YA 112 | R | TTTTTC | AAAAAG |  |  |  |  |  |  |  |  |
| YA 113 | S | TTTTTT | AAAAAG |  |  |  |  |  |  |  |  |
| YA 114 | R | TTTTTC | AAAAAG |  |  |  |  |  |  |  |  |
| YA 115 | S | TTTTTT | AAAAAA |  |  |  |  |  |  |  |  |
| YA 116 | S | TTTTTT | AAAAAA |  |  |  |  |  |  |  |  |
| YA 117 | S | TTTTTT | AAAAAA |  |  |  |  |  |  |  |  |
| YA 118 | R | TTTTTC | AAAAAG |  |  |  |  |  |  |  |  |
| YA 119 | R | TTTTTC | AAAAAG |  |  |  |  |  |  |  |  |
| YA 120 | S | TTTTTT | AAAAAA |  |  |  |  |  |  |  |  |
| YA 121 | S | TTTTTT | AAAAAA |  |  |  |  |  |  |  |  |
| YA 122 | S | TTTTTT | AAAAAA |  |  |  |  |  |  |  |  |
| YA 123 | S | TTTTTT | AAAAAA |  |  |  |  |  |  |  |  |
| YA 124 | S | TTTTTT | AAAAAA |  |  |  |  |  |  |  |  |
| YA 125 | S | TTTTTT | AAAAAA |  |  |  |  |  |  |  |  |
| YA 126 | R | TTTTTC | AAAAAG |  |  |  |  |  |  |  |  |
| YA 127 | S | TTTTTT | AAAAAG |  |  |  |  |  |  |  |  |
| YA 128 | R | TTTTTC | AAAAAG |  |  |  |  |  |  |  |  |
| YA 129 | S | TTTTTT | AAAAAG |  |  |  |  |  |  |  |  |
| YA 130 | S | TTTTTT | AAAAAA |  |  |  |  |  |  |  |  |
| YA 131 | S | TTTTTT | AAAAAA |  |  |  |  |  |  |  |  |
| YA 132 | S | TTTTTT | AAAAAA |  |  |  |  |  |  |  |  |
| YA 133 | S | TTTTTT | AAAAAA |  |  |  |  |  |  |  |  |
| YA 134 | R | TTTTTC | AAAAAG |  |  |  |  |  |  |  |  |
| YA 135 | S | TTTTTT | AAAAAA |  |  |  |  |  |  |  |  |
| YA 136 | R | TTTTTC | AAAAAG |  |  |  |  |  |  |  |  |
| YA 137 | R | TTTTTC | AAAAAG |  |  |  |  |  |  |  |  |
| YA 138 | S | TTTTTT | AAAAAA |  |  |  |  |  |  |  |  |
| YA 139 | R | TTTTTT | AAAAAG |  |  |  |  |  |  |  |  |
| YA 140 | R | TTTTTC | AAAAAG |  |  |  |  |  |  |  |  |
| YA 141 | R | TTTTTC | AAAAAG |  |  |  |  |  |  |  |  |
| YA 142 | R | TTTTTC | AAAAAG |  |  |  |  |  |  |  |  |
| YA 143 | S | TTTTTT | AAAAAA |  |  |  |  |  |  |  |  |
| YA 144 | S | TTTTTT | AAAAAA |  |  |  |  |  |  |  |  |
| YA 145 | R | TTTTTC | AAAAAG |  |  |  |  |  |  |  |  |
| YA 146 | S | TTTTTT | AAAAAA |  |  |  |  |  |  |  |  |
| YA 147 | R | TTTTTC | AAAAAG |  |  |  |  |  |  |  |  |
| YA 148 | R | TTTTTC | AAAAAG |  |  |  |  |  |  |  |  |
| YA 149 | R | TTTTTC | AAAAAG |  |  |  |  |  |  |  |  |
| YA 150 | R | TTTTTC | AAAAAG |  |  |  |  |  |  |  |  |
| YA 151 | R | TTTTTC | AAAAAG |  |  |  |  |  |  |  |  |
| YA 152 | S | TTTTTT | AAAAAA |  |  |  |  |  |  |  |  |
| YA 153 | R | TTTTTC | AAAAAG |  |  |  |  |  |  |  |  |
| YA 154 | R | TTTTTC | AAAAAG |  |  |  |  |  |  |  |  |
| YA 155 | S | TTTTTT | AAAAAA |  |  |  |  |  |  |  |  |
| YA 156 | S | TTTTTT | AAAAAA |  |  |  |  |  |  |  |  |
| YA 157 | S | TTTTTT | AAAAAA |  |  |  |  |  |  |  |  |
| YA 158 | S | TTTTTT | AAAAAA |  |  |  |  |  |  |  |  |
| YA 159 | S | TTTTTT | AAAAAA |  |  |  |  |  |  |  |  |
| YA 160 | R | TTTTTC | AAAAAG |  |  |  |  |  |  |  |  |
| YA 161 | R | TTTTTC | AAAAAG |  |  |  |  |  |  |  |  |
| YA 162 | R | TTTTTC | AAAAAG |  |  |  |  |  |  |  |  |
| YA 163 | S | TTTTTT | AAAAAA |  |  |  |  |  |  |  |  |
| YA 164 | S | TTTTTT | AAAAAA |  |  |  |  |  |  |  |  |
| YA 165 | S | TTTTTT | AAAAAA |  |  |  |  |  |  |  |  |
| YA 166 | R | TTTTTC | AAAAAG |  |  |  |  |  |  |  |  |
| YA 167 | R | TTTTTC | AAAAAG |  |  |  |  |  |  |  |  |
| YA 168 | S | TTTTTT | AAAAAA |  |  |  |  |  |  |  |  |
| YA 169 | R | TTTTTC | AAAAAG |  |  |  |  |  |  |  |  |

^a^Plants that showed no telia were scored as resistant. Plants were scored as susceptible when at least one telium was observed. P. horiana resistance was examined by three separate inoculations using the isolate TS.

^b^The genotypes of SCSE_SC004884.1_65872 in resistant ‘Ariesu’ and susceptible ‘Yellow Queen’ were TTTTTC and TTTTTT, respectively. The genotypes of SCSE_SC000716.1_75925 in resistant ‘Ariesu’ and susceptible ‘Yellow Queen’ were AAAAAG and AAAAAA, respectively.
