# Supplementary material for: A genome-wide association and fine-mapping study of white rust resistance in hexaploid chrysanthemum cultivars with a wild diploid reference genome
Source: Hortic Res. 2022 Aug 3;9:uhac170. doi: 10.1093/hr/uhac170 (PMC9613985; doi:10.1093/hr/uhac170)
Supplement: Web_Material_uhac170 [file web_material_uhac170.zip › SupplentaryDataTables_r1.doc]

Supplementary Data Tables

| **Supplementary Table S1**. Cultivars used, including plant type and source | | |
| --- | --- | --- |
| Cultivar | Type | Source |
| Ariesu | Spray type | National Federation of Agricultural Cooperative Associations |
| Yellow Queen | Spray type | Genebank Project (NARO), Sumitomo et al. (2021) |
| Bunmei | Standard type | Genebank Project (NARO), Yamaguchi (1981) |
| Jimba | Standard type | Genebank Project (NARO), Iwai et al. (2009) |
| Kanamarufuji | Standard type | Genebank Project (NARO), Iwai et al. (2009) |
| Kankohbai | Spray type | Genebank Project (NARO), Yamaguchi (1981) |
| Kanran | Spray type | Genebank Project (NARO), Yamaguchi (1981) |
| Kanseisetsu | Standard type | Genebank Project (NARO), Iwai et al. (2009) |
| Kinkou | Standard type | Genebank Project (NARO), Yamaguchi (1981) |
| Kyura Kids | Spray type | Kagoshima Prefectural Institute for Agricultural Development |
| Kyura Shusa | Spray type | Kagoshima Prefectural Institute for Agricultural Development |
| Meimon | Standard type | Genebank Project (NARO), Iwai et al. (2009) |
| Moze Cute | Spray type | Kagoshima Prefectural Institute for Agricultural Development |
| Moze Frame | Spray type | Kagoshima Prefectural Institute for Agricultural Development |
| Otomezakura | Standard type | Genebank Project (NARO), Yamaguchi (1981) |
| Penny Lane | Spray type | Genebank Project (NARO), Park et al. (2014) |
| Purinsesu | Standard type | Genebank Project (NARO), Yamaguchi (1981) |
| Refour | Spray type | Genebank Project (NARO), De Jong and Rademaker (1986) |
| Sanyo-ougon | Standard type | Genebank Project (NARO), Iwai et al. (2009) |
| Seikonohikari | Standard type | Genebank Project (NARO), Yamaguchi (1981) |
| Seiko-no-mine | Standard type | Genebank Project (NARO), Iwai et al. (2009) |
| Shuho-no-chikara | Standard type | Genebank Project (NARO), Yamaguchi (1981) |
| Shuho-no-kokoro | Standard type | Genebank Project (NARO), Iwai et al. (2009) |
| Snowdon | Spray type | Genebank Project (NARO), Martin and Firman (1970) |
| Southern Pegasus | Spray type | Kagoshima Prefectural Institute for Agricultural Development, Sumitomo et al. (2021) |
| Southern Shell | Spray type | Kagoshima Prefectural Institute for Agricultural Development |
| TM Miruku | Spray type | Genebank Project (NARO) |
| Westland Pink | Spray type | Genebank Project (NARO) |
| SEI01 | Spray type | Inochio Seikoen |
| SEI02 | Spray type | Inochio Seikoen |
| SEI03 | Spray type | Inochio Seikoen |
| SEI04 | Spray type | Inochio Seikoen |
| SEI05 | Spray type | Inochio Seikoen |
| SEI06 | Spray type | Inochio Seikoen |
| SEI07 | Spray type | Inochio Seikoen |
| SEI08 | Spray type | Inochio Seikoen |
| SEI09 | Spray type | Inochio Seikoen |
| SEI10 | Spray type | Inochio Seikoen |
| SEI11 | Spray type | Inochio Seikoen |
| SEI12 | Spray type | Inochio Seikoen |
| SEI13 | Spray type | Inochio Seikoen |
| SEI14 | Spray type | Inochio Seikoen |
| SEI15 | Standard type | Inochio Seikoen |
| SEI16 | Standard type | Inochio Seikoen |
| SEI17 | Standard type | Inochio Seikoen |
| SEI18 | Standard type | Inochio Seikoen |

| **Supplementary Table S2.** Isolates of *P. horiana* with isolate code, origin, and source | | |
| --- | --- | --- |
| Isolate code | Origin in Japan | Source |
| TS | Tsukuba | NARO |
| NA | Nara | Nara Prefecture Agricultural Research and Development Center |
| AK | Akita | Akita Agricultural Experiment Station |
| IB | Ibaraki | Ibaraki Horticultural Research Institute |
| TO1 | Toyama | Cut flower grower |
| TO2 | Toyama | Cut flower grower |
| TO3 | Toyama | Cut flower grower |
|  |  |  |

| **Supplementary Table S3.** Primers used in PCR | | |  |  |
| --- | --- | --- | --- | --- |
|  |  | Sequence (5′ → 3′) |  | Amplicon  size (bp) |
| SNP | Allele | Forward | Reverse |  |
| SCSE_SC004884.1_65872 | Resistant | GGGCAACACCCTTTAATCC | TTGAACAACCCCATCAAGA | 171 |
|  | Susceptible | GGGCAACACCCTTTAATCC | TTGAACAACCACATCAAGT | 171 |
| SCSE_SC000716.1_75925 | Resistant | AAACCAATCACTGGCAAGCAGTT | TTCTGATTTGATGTCCCCCCT | 168 |
|  | Susceptible | CCAATCACTGGCAGGCAGTC | TTCTGATTTGATGTCTCCCCG | 165 |
| Cse2.0_LG9_46170750^T^ | Resistant | CTCCTACTCTACAAATGGCGAT | CGACCTAATAATATTGGGTGAAG | 76 |
|  | Susceptible | CTCCTACTCTACAAATGGCGGC | CGACCTAATAATATTGGTCGTAT | 76 |
| Cse2.0_LG9_46476925^C^ | Resistant | GTTATAAGTTATCTTGATTAGCATAAAC | GCTATTTAATAGGTACCGCAT | 172 |
|  | Susceptible | GTTATAAGTTATCTTGATTAGCATAAAT | GCTATTTAATAGGTACCGCAG | 172 |
| Cse2.0_LG9_47009004^G^ | Resistant | CTCAAAACAAAAGCTGAATATGG | GAATTCATGTAAGAGACATTTGACA | 209 |
|  | Susceptible | CTCAAAACAAAAGCTGAATATGA | GAATTCATGTAAGAGACATTTGACG | 209 |
| Cse2.0_LG9_47483611^T^ | Resistant | AGAAAATCTTTCGCTTCATTTTCTT | ATTTGCCCAAATGAGGTAAAA | 127 |
|  | Susceptible | AGAAAATCTTTCGCTTCATTTTCTG | ATTTGCCCAAATGAGGTAAAG | 127 |
| Cse2.0_LG9_48084985^C^ | Resistant | CACCGCTGACTATAAAAACTCATTC | GTGTAAAGGGATCTTTGCTTGTC | 70 |
|  | Susceptible | CACCGCTGACTATAAAAACTCACTT | GTGTAAAGGGATCTTTGCTTGTG | 70 |
| Cse2.0_LG9_48453417^A^ | Resistant | ATTTCGCCCACCCTCAAA | ACCCTTTTGTGGAATTTAGCAAC | 147 |
|  | Susceptible | ATTTCGCCCACCCTCAAG | ACCCTTTTGTGGAATTTAGCAAG | 147 |
| Cse2.0_LG9_49032201^A^ | Resistant | TAACATCCTCCAATTCCCTAATCA | TGAGGGTGCACTCAGGCA | 62 |
|  | Susceptible | TAACATCCTCCAATTCCCTAATCG | TGAGGGTGCACTCAGGCT | 62 |
| Cse2.0_LG9_50042598^A^ | Resistant | GTGAGAAGAAGGCTCTCTCAA | CCATTCCCAGCTAAAAATCAG | 101 |
|  | Susceptible | ATGAGAAGAAGGCTCTCTCAG | CCATTCCCAGCTAAAAATCAC | 101 |
|  |  |  |  |  |

| **Supplementary Table S4.** General linear model association analysis showing 82 single nucleotide polymorphisms associated with *P. horiana* resistance | | | | | |
| --- | --- | --- | --- | --- | --- |
|  |  |  |  | Parental genotype | |
| SNP marker name | *p*-value | Major allele | Minor allele | Ariesu | Yellow Queen |
| SCSE_SC004884.1_65872 | 1.12 × 10^-113^ | T | C | TTTTTC | TTTTTT |
| SCSE_SC000716.1_75925 | 2.76 × 10^-106^ | A | G | AAAAAG | AAAAAA |
| SCSE_SC004884.1_65864 | 6.92 × 10^-105^ | A | T | AAAAAT | AAAAAA |
| SCSE_SC000716.1_75968 | 1.93 × 10^-102^ | T | C | TTTTTC | TTTTTT |
| SCSE_SC021543.1_28574 | 7.74 × 10^-96^ | G | T | GGGGGT | GGGGGG |
| SCSE_SC000716.1_76105 | 1.84 × 10^-89^ | C | T | CCCCCT | CCCCCC |
| SCSE_SC009962.1_74877 | 1.38 × 10^-77^ | G | A | GGGGGA | GGGGGG |
| SCSE_SC009853.1_24217 | 3.54 × 10^-77^ | A | G | AAAAAG | AAAAAA |
| SCSE_SC013456.1_34100 | 1.08 × 10^-60^ | G | A | GGGGGA | GGGGGG |
| SCSE_SC027670.1_8862 | 6.65 × 10^-53^ | C | A | CCCCCA | CCCCCC |
| SCSE_SC030319.1_18939 | 2.94 × 10^-52^ | A | C | AAAAAC | AAAAAA |
| SCSE_SC063616.1_5180 | 3.36 × 10^-49^ | G | C | GGGGGC | GGGGGG |
| SCSE_SC027465.1_25683 | 1.23 × 10^-47^ | A | T | AAAAAT | AAAAAA |
| SCSE_SC027465.1_25675 | 1.33 × 10^-47^ | G | A | GGGGGA | GGGGGG |
| SCSE_SC027465.1_25802 | 1.33 × 10^-47^ | T | C | TTTTTC | TTTTTT |
| SCSE_SC019720.1_34075 | 2.30 × 10^-47^ | A | G | AAAAAG | AAAAAA |
| SCSE_SC006585.1_340 | 2.47 × 10^-46^ | T | C | TTTTTC | TTTTTT |
| SCSE_SC006585.1_486 | 2.72 × 10^-46^ | G | T | GGGGGT | GGGGGG |
| SCSE_SC007072.1_57135 | 6.88 × 10^-46^ | A | G | AAAAAG | AAAAAA |
| SCSE_SC019720.1_34076 | 2.19 × 10^-43^ | C | T | CCCCCT | CCCCCC |
| SCSE_SC027465.1_25686 | 9.22 × 10^-43^ | T | C | TTTTTC | TTTTTT |
| SCSE_SC006585.1_318 | 1.09 × 10^-42^ | C | T | CCCCCT | CCCCCC |
| SCSE_SC000014.1_49514 | 4.65 × 10^-42^ | T | C | TTTTTC | TTTTTT |
| SCSE_SC000646.1_7799 | 9.61 × 10^-42^ | T | C | TTTTTC | TTTTTT |
| SCSE_SC019047.1_18416 | 1.93 × 10^-41^ | A | T | AAAAAT | AAAAAA |
| SCSE_SC019047.1_18450 | 5.15 × 10^-41^ | C | G | CCCCCG | CCCCCC |
| SCSE_SC027465.1_25687 | 1.83 × 10^-40^ | G | A | GGGGGA | GGGGGG |
| SCSE_SC007072.1_57105 | 3.56 × 10^-40^ | C | T | CCCCCT | CCCCCC |
| SCSE_SC001053.1_28146 | 6.46 × 10^-40^ | A | G | AAAAAG | AAAAAA |
| SCSE_SC003564.1_46888 | 2.81 × 10^-39^ | C | T | CCCCCT | CCCCCC |
| SCSE_SC005247.1_49114 | 9.31 × 10^-38^ | A | G | AAAAAG | AAAAAA |
| SCSE_SC005247.1_49174 | 9.31 × 10^-38^ | A | G | AAAAAG | AAAAAA |
| SCSE_SC005206.1_69368 | 2.60 × 10^-37^ | C | A | CCCCCA | CCCCCC |
| SCSE_SC003615.1_54983 | 2.83 × 10^-37^ | C | G | CCCCCG | CCCCCC |
| SCSE_SC006860.1_61125 | 3.92 × 10^-37^ | G | T | GGGGGT | GGGGGG |
| SCSE_SC000014.1_49305 | 6.42 × 10^-37^ | A | T | AAAAAT | AAAAAA |
| SCSE_SC007072.1_57022 | 2.12 × 10^-36^ | A | C | AAAAAC | AAAAAA |
| SCSE_SC020563.1_10774 | 5.97 × 10^-36^ | T | A | TTTTTA | TTTTTT |
| SCSE_SC004830.1_51550 | 1.00 × 10^-35^ | T | C | TTTTTC | TTTTTT |
| SCSE_SC001648.1_102507 | 1.03 × 10^-35^ | C | T | CCCCCT | CCCCCC |
| SCSE_SC004830.1_41426 | 5.96 × 10^-35^ | T | A | TTTTTA | TTTTTT |
| SCSE_SC001648.1_102440 | 1.26 × 10^-33^ | T | C | TTTTTC | TTTTTT |
| SCSE_SC001648.1_102459 | 3.44 × 10^-32^ | A | T | AAAAAT | AAAAAA |
| SCSE_SC001648.1_102137 | 9.61 × 10^-32^ | C | A | CCCCCA | CCCCCC |
| SCSE_SC003564.1_46901 | 1.98 × 10^-31^ | G | A | GGGGGA | GGGGGG |
| SCSE_SC001648.1_102111 | 2.37 × 10^-31^ | G | A | GGGGGA | GGGGGG |
| SCSE_SC007981.1_53521 | 2.94 × 10^-31^ | C | A | CCCCCA | CCCCCC |
| SCSE_SC003615.1_55058 | 9.88 × 10^-31^ | C | T | CCCCCT | CCCCCC |
| SCSE_SC000040.1_29437 | 1.21 × 10^-30^ | G | A | GGGGGA | GGGGGG |
| SCSE_SC027465.1_25765 | 3.37 × 10^-30^ | G | A | GGGGGA | GGGGGG |
| SCSE_SC011825.1_27391 | 2.59 × 10^-29^ | C | T | CCCCCT | CCCCCC |
| SCSE_SC000014.1_49252 | 8.38 × 10^-29^ | A | G | AAAAAG | AAAAAA |
| SCSE_SC008263.1_46976 | 1.38 × 10^-28^ | G | A | GGGGGA | GGGGGG |
| SCSE_SC008263.1_46961 | 8.47 × 10^-28^ | C | T | CCCCCT | CCCCCC |
| SCSE_SC032041.1_7558 | 9.34 × 10^-28^ | C | G | CCCCCG | CCCCCC |
| SCSE_SC032041.1_7590 | 6.81 × 10^-27^ | T | G | TTTTTG | TTTTTT |
| SCSE_SC032041.1_7555 | 4.93 × 10^-26^ | A | G | AAAAAG | AAAAAA |
| SCSE_SC000305.1_86815 | 4.61 × 10^-24^ | T | C | TTTTTC | TTTTTT |
| SCSE_SC184532.1_530 | 5.08 × 10^-24^ | T | C | TTTTTC | TTTTTT |
| SCSE_SC027591.1_18577 | 8.73 × 10^-24^ | C | G | CCCCCG | CCCCCC |
| SCSE_SC215427.1_216 | 1.33 × 10^-23^ | T | C | TTTTTC | TTTTTT |
| SCSE_SC005247.1_48223 | 1.62 × 10^-23^ | A | G | AAAAAG | AAAAAA |
| SCSE_SC007237.1_6431 | 1.63 × 10^-23^ | C | A | CCCCCA | CCCCCC |
| SCSE_SC007237.1_6444 | 3.30 × 10^-22^ | C | A | CCCCCA | CCCCCC |
| SCSE_SC018258.1_24241 | 4.76 × 10^-22^ | G | A | GGGGGA | GGGGGG |
| SCSE_SC004750.1_46374 | 1.74 × 10^-20^ | T | G | TTTTTG | TTTTTT |
| SCSE_SC008588.1_44212 | 2.02 × 10^-20^ | T | C | TTTTTC | TTTTTT |
| SCSE_SC009962.1_75067 | 2.25 × 10^-19^ | A | G | AAAAAG | AAAAAA |
| SCSE_SC000639.1_56008 | 1.72 × 10^-18^ | T | C | TTTTTC | TTTTTT |
| SCSE_SC005960.1_28750 | 2.53 × 10^-18^ | G | T | GGGGGT | GGGGGG |
| SCSE_SC000639.1_56021 | 5.27 × 10^-18^ | G | T | GGGGGT | GGGGGG |
| SCSE_SC000639.1_56003 | 1.58 × 10^-17^ | T | A | TTTTTA | TTTTTT |
| SCSE_SC018555.1_12878 | 9.82 × 10^-16^ | C | T | CCCCCT | CCCCCC |
| SCSE_SC018555.1_12881 | 9.82 × 10^-16^ | A | G | AAAAAG | AAAAAA |
| SCSE_SC004750.1_46187 | 2.85 × 10^-15^ | C | T | CCCCCT | CCCCCC |
| SCSE_SC012603.1_17638 | 2.41 × 10^-11^ | C | T | CCCCCT | CCCCCC |
| SCSE_SC209696.1_345 | 6.94 × 10^-10^ | T | A | TTTTTA | TTTTTT |
| SCSE_SC209696.1_426 | 1.48 × 10^-9^ | A | G | AAAAAG | AAAAAA |
| SCSE_SC015035.1_29736 | 1.59 × 10^-9^ | C | T | CCCCCT | CCCCCC |
| SCSE_SC008299.1_82602 | 6.07 × 10^-9^ | T | A | TTTTTA | TTTTTT |
| SCSE_SC005396.1_52990 | 3.87 × 10^-7^ | T | C | TTTTTC | TTTTTT |
| SCSE_SC034939.1_14041 | 7.62 × 10^-7^ | A | C | AAAAAC | AAAAAA |

| **Supplementary Table S5.** Statistics of whole genome assembly of ‘Ariesu’ and ‘Yellow Queen’ | | | |
| --- | --- | --- | --- |
|  |  | Ariesu | Yellow Queen |
| All | Number of sequences | 101,290,730 | 97,821,561 |
|  | Total length (bp) | 21,001,896,460 | 20,449,854,573 |
|  | Average length (bp) | 207 | 209 |
|  | Max length (bp) | 118,120 | 102,803 |
|  | Min length (bp) | 102 | 102 |
|  | N50 length (bp) | 203 | 203 |
|  | A | 6,330,281,009 | 6,185,411,514 |
|  | T | 6,085,153,303 | 5,948,226,939 |
|  | G | 3,511,045,572 | 3,430,505,831 |
|  | C | 3,652,108,170 | 3,566,227,569 |
|  | n | 0 | 0 |
|  | N | 1,423,308,406 | 1,319,482,720 |
|  | Total (ATGC) | 19,578,588,054 | 19,130,371,853 |
|  | GC% (ATGC) | 36.6 | 36.6 |
| ≥500 b | Number of sequences | 3,743,825 | 3,521,557 |
|  | Total length (bp) | 8,061,183,967 | 7,986,482,176 |
|  | Average length (bp) | 2,153 | 2,268 |
